# Letter to the editor: Comments on - Regulation of habenular G-protein gamma 8 on learning and memory via modulation of the central acetylcholine system

**DOI:** 10.1038/s41380-022-01451-8

**Published:** 2022-02-03

**Authors:** Andrew J. Lawrence

**Affiliations:** 1 Florey Institute of Neuroscience & Mental Health, University of Melbourne, Parkville, VIC 3052 Australia; 2 Florey Department of Neuroscience & Mental Health, University of Melbourne, Parkville, VIC 3052 Australia

**Keywords:** Neuroscience, Molecular biology

G-protein gamma 8 (Gng8), sometimes referred to as γ8(olf), belongs to the family of gamma subunits of heterotrimeric G proteins [1, 2]. While initial studies suggested this subunit was restricted to olfactory and vomeronasal systems [1], comprehensive mapping suggests that γ8(olf) is more widely expressed in a number of rodent brain regions, although enriched in the habenula [2]. Given the location of *Gng8* within 19q13.32 from where deletions have been associated with intellectual disability [3], the question arises as to whether Gng8 has any role in regulating cognitive function. In this context, Lee et al. recently reported a comprehensive study, including zebra fish and a novel *Gng8* knockout mouse highlighting a putative role for Gng8 in aspects of spatial and long-term memory [4]. While I applaud the authors in their comprehensive and multidisciplinary approach, there are some points worthy of discussion.

Characterisation of the *Gng8* KO mice and behavioural analysis in the Morris Water maze showed intact spatial learning but attenuated spatial memory when mice were tested in the absence of the escape platform (probe trial). Close scrutiny of Fig. 3d however suggests all mice are actually avoiding the target quadrant (only ~6% WT and ~4% KO time in target quadrant) after spatial learning—this is most unusual. A similar experiment is repeated (Fig. 5) that includes pharmacological interventions. In this dataset for the probe trial (Fig. 5b), the WT vehicle show ~30% time in the target quadrant, with *Gng8* KO mice showing reduced time (~20%). It is not clear why the two datasets (Fig. 3d vs Fig. 5b) are so different from each other.

Focusing on the habenula and its output targets, the authors showed that in *Gng8* KO mice acetylcholine (ACh) levels were reduced in the medial habenula (mHb) and choline acetyltransferase (ChAT) was reduced in both the mHb and the interpeduncular nucleus (IPN), especially IPR and IPC. The team subsequently demonstrated impaired high frequency stimulation induced hippocampal long-term potentiation suggesting that “decrease in cholinergic activity of the MHb–IPN pathway in *Gng8* KO mice could affect hippocampal synaptic plasticity”. While on the surface this appears a perfectly reasonable hypothesis, the devil is in the detail. The link between the mHb – IPN pathway and the hippocampus (HC) was suggested to be via a direct IPN – HC pathway. Critically, the authors stated “To examine cholinergic projection from the IPN to the HC, FG retrograde tracer was injected into the HC, and immunoreactivity of FG and ChAT in the IPN was quantified. Manders’ coefficient was 0.4438, and Costes *P* value was 0.9863 (Fig. 4c). These data indicated that 44.38% of IPN–HC efferents are cholinergic neurons”. Usually such a conclusion would also be supported by cell counts. Moreover, in the discussion the authors state “Although the IPN sends minor efferent to the HC…, 56.30% of these IPN efferents were cholinergic…”.

Cholingeric neurons in the mHb innervate the IPN via the fasciculus retroflexus (FR) [as reviewed in [5]. Lee et al. are also suggesting that cholinergic neurons project from the IPN to the HC. It has been noted for decades that the ChAT staining in the IPN is located in “small fibres and in punctate structures suggestive of axon terminals” [6]. As noted by Eckenrode et al. “No ChAT-stained perikarya were seen in the IPN, even after colchicine treatment” [7]. Moreover, the latter study further demonstrated that bilateral lesions of FR “eliminated all ChAT from the IPN”, supporting an extrinsic source of cholinergic input, rather than it being intrinsic to the IPN. Another protein required for cholinergic transmission is the vesicular acetylcholine transporter (VAChT) and staining for VAChT occurs in axons and terminals within the IPN [8]. However, the same authors failed to find expression of the mRNA encoding either ChAT or VAChT within the IPN, or immunostaining in cell bodies [8].

Indeed, evidence for cholinergic neuronal cell bodies in IPR or IPC (the focus of the current study) is therefore lacking. For transparency, in rat brain, there is a report of VAChT in a scattering of cells in the lateral aspects of IPN [9], although this is not universally agreed upon [10]. Figure 4c appears to show retrogradely labelled cells scattered around a plexus of ChAT fibres, rather than within cholinergic neurons in the IPN. One possible explanation for the authors’ interpretation that cells in IPN projecting to HC are cholinergic may lie in the use of analysis of fluorescence intensity and Manders Coefficient to determine colocalization. While this approach has been used extensively, recent careful studies suggest this method is “not suitable for making measurements of colocalization either by correlation or co-occurrence…or as a summary measurement” [11].

From the data presented, it is therefore difficult support the rather definitive conclusion that “44.38% of IPN–HC efferents are cholinergic neurons”. It is however distinctly possible that the cells in the IPN that project to the HC receive a cholinergic input from mHb, and reduced cholinergic transmission at mHb—IPN synapses could perceivably impact the activity of IPN—HC neurons and thereby contribute to the phenotype observed. Nevertheless, in rats Gng8 is not restricted in expression to the habenula—interpeduncular system [2] and therefore lack of Gng8 in other brain regions (such as HC) may also contribute to the behavioural and electrophysiological phenotypes reported.
